# Association of Clinical Frailty Scores With Hospital Readmission for Falls After Index Admission for Trauma-Related Injury

**DOI:** 10.1001/jamanetworkopen.2019.12409

**Published:** 2019-10-02

**Authors:** Victor H. Hatcher, Colette Galet, Michele Lilienthal, Dionne A. Skeete, Kathleen S. Romanowski

**Affiliations:** 1 Carver College of Medicine, University of Iowa, Iowa City; 2 Division of Acute Care Surgery, Department of Surgery, Carver College of Medicine, University of Iowa, Iowa City; 3 Department of Surgery, University of California, Davis and Shriners Hospitals for Children, Northern California, Sacramento, California

## Abstract

**Importance:**

Falls have been associated with morbidity and mortality in elderly patients. Assessment of frailty at hospital admission may help health care professionals evaluate fall risk in patients with trauma-related injury.

**Objective:**

To determine whether frailty assessed using the Canadian Study of Health and Aging Clinical Frailty Scale is associated with readmission for falls after index admission for trauma-related injury in patients aged 50 years and older.

**Design, Setting, and Participants:**

This retrospective cohort study reviewed the medical records of 804 patients aged 50 years and older with trauma-related injury who were admitted to the University of Iowa Hospitals and Clinics between July 1, 2010, and June 30, 2015. Records were reviewed from May 30 to August 1, 2017, and patient demographics, admission data, injury severity scores, history of falls, and postindex readmission data for ground-level falls were recorded. Frailty scores were calculated using the Canadian Study of Health and Aging Clinical Frailty Scale. Patients with a score of 5 or higher were classified as frail.

**Main Outcomes and Measures:**

Frailty assessed using the Canadian Study of Health and Aging Clinical Frailty Scale and readmission for falls after index admission for trauma-related injury.

**Results:**

A total of 804 patients with trauma-related injury were included in the study. The mean (SD) age was 70 (13.4) years; 744 patients (93.4%) were white, and 380 (47.3%) were men. Among the total population, the mortality rate was 3.7%; 255 patients (31.7%) were classified as frail and 549 (68.3%) as nonfrail. The mean (SD) injury severity score was 9.8 (7.9), and the score was similar between frail and nonfrail patients. Of 255 frail patients, 179 (70.2%) were women, and frail patients were significantly older than nonfrail patients (mean [SD], 79.2 [12.1] years vs 66.2 [11.9] years, respectively; *P* < .001). The percentages of frail patients presenting to the hospital with a history of falls and readmitted for falls after index admission were higher than those of nonfrail patients (63 [24.8%] vs 53 [9.6%] and 55 [21.6%] vs 58 [10.6%], respectively; both *P* < .001). Frailty was associated with discharge to the home with health care (odds ratio [OR], 4.82; 95% CI, 2.10-11.01; *P* < .001), to a skilled nursing facility (OR, 5.47; 95% CI, 3.40-8.80; *P* < .001), and to a hospice care facility (OR, 8.47; 95% CI, 2.09-34.42; *P* = .003) compared with discharge to the home with self-care. Frailty was also associated with readmission for falls after index admission (OR, 2.26; 95% CI, 1.39-3.66; *P* = .001) and the number of falls within 1 year after index admission (OR, 1.32; 95% CI, 1.04-1.67; *P* = .02) compared with nonfrailty. The frailty analysis was controlled for age, body mass index, sex, and falls at index admission.

**Conclusions and Relevance:**

Measurement of frailty at hospital admission may be an effective tool to assess fall risk and discharge disposition among patients with trauma-related injury aged 50 years and older.

## Key Points

**Question:**

Is frailty assessed using the Canadian Study of Health and Aging Clinical Frailty Scale associated with hospital readmission for falls after index admission for trauma-related injury?

**Findings:**

In this cohort study of 804 patients aged 50 years and older with trauma-related injury, frailty was more likely to be associated with readmission for falls after index admission and discharge disposition to a skilled nursing facility compared with nonfrailty.

**Meaning:**

Assessment of frailty at hospital admission may provide an effective tool to evaluate fall risk and the likelihood of discharge disposition to a skilled nursing facility among patients with trauma-related injury aged 50 years and older, and may provide useful information for discharge planning by evaluating the need for referrals to fall prevention programs among at-risk patients and by estimating potential health care costs.

## Teaser

This cohort study assesses whether clinical frailty scores are associated with readmission for falls after index admission for trauma-related injury among patients aged 50 years and older.

## Introduction

The number of elderly adults in the United States is now larger than it has ever been. Census data from 2010 reveal that 40.4 million people are aged 65 years and older. This number represents a 5.3% increase from the 2000 census, and the elderly population is estimated to more than double between 2012 and 2060.^1^ Fall-related injuries are associated with morbidity and mortality in elderly people. Approximately 30% of community-dwelling adults aged 65 years and older experience 1 or more falls each year.^2^ Falls were the leading cause of nonfatal trauma-related injury in patients aged 65 years and older in 2015, making up 63.8% of injuries.^3^ An estimated 23% to 30% of elderly individuals have experienced falls; however, less than half of them reported these incidents to their physicians.^4^ Falls have been reported to often lead to a fear of falling or loss of confidence, which may result in a decline in overall activity, leading to decreased strength, balance, and mobility; cumulating in decreased functional ability and loss of independence.^5,6^

A recent analysis of the Healthcare Cost and Utilization Project’s Nationwide Readmissions Database reported that deaths and admissions for fall-related injuries in the elderly population are steadily increasing. Moreover, the incidence of readmission for a subsequent fall is increasing.^7^ With the rising population of elderly individuals in the United States, health care professionals and institutions face many new challenges in the care of elderly patients.

Age alone is not a sufficient factor in estimating physiological fitness in the elderly population; thus, many scales have been developed to measure patient frailty. However, many of these scales have been reported to be difficult to administer. Rockwood et al^8^ created and validated the Canadian Study of Health and Aging Clinical Frailty Scale (CSHA CFS), which was adapted from a larger 70-item frailty index.^8^ The CSHA CFS is a 7-point clinical opinion scale that has been reported to have good criterion validity, with reasonable construct validity and a dose-response effect in relation to a 5-year association with death or admission to an institutional facility.^8^ This simple clinical opinion scale has been validated for estimating the mortality and institutionalization of patients over age 65 receiving care from internal medicine clinicians.^9^ The CSHA CFS allows clinicians to assess a patient’s physiological status based on comorbidities, therapeutic management, and the assistance needed to accomplish the activities of daily living. Using these criteria, a clinical frailty score is assigned to each patient. For the purposes of this study, any medical problem (including substance abuse and mental health diagnoses) that was listed in the patient’s medical history was considered a comorbidity. The severity of the comorbidity was determined by examining the number of medications required to control the comorbidity, the level to which control was achieved (eg, glycated hemoglobin), and the number of comorbidities present.

Masud et al^10^ used the CSHA CFS to examine the association of preinjury physical condition with mortality and outcomes among patients with burns covering more than 10% of their total body surface; the study reported that high frailty scores (ie, poor preinjury physical condition) were associated with a higher likelihood of mortality in elderly patients with burn injuries. Romanowski et al^11,12^ replicated these results using no burn-size cutoff, reporting that CSHA CFS scores were associated with the likelihood of mortality and discharge to a skilled nursing facility (SNF) not only in patients with burn injuries but in those with trauma-related injuries. The simplicity and ease of use of the CSHA CFS scale make it ideal for the clinical setting. Yet, its applicability to detecting fall risk is unknown. We hypothesized that frailty assessed using the CSHA CFS would be associated with readmission for falls after index admission and with the number of falls within 1 year after index admission. The CSHA CFS was used to measure frailty in patients with trauma-related injury aged 50 years and older and to assess whether frailty was associated with readmission for falls and the number of falls within 1 year after index admission.

## Methods

### Study Design

Patients aged 50 years and older admitted for any trauma-related injury, with the exception of a burn injury, from July 1, 2010, to June 30, 2015, and with a zip code in Johnson County, Iowa, were included in the study because they were considered more likely to represent the patients admitted to the University of Iowa Hospitals and Clinics, allowing us to assess readmission for falls after index admission. Data collection was performed between May 30 and August 1, 2017, and data were examined for readmission for falls within 1 year after index admission for all patients in the sample. This study followed the Strengthening the Reporting of Observational Studies in Epidemiology (STROBE) reporting guideline for cohort studies, and it was approved by the institutional review board of the University of Iowa with a waiver of consent.

### Data Collection

We identified clinical variables from the electronic medical record using database queries and manual data collection performed by 2 of us (V.H.H. and K.S.R.). Data were collected from the University of Iowa Hospitals and Clinics trauma registry and electronic medical record, including demographics (age, sex, race, comorbidities, psychiatric conditions, substance abuse history, and body mass index [BMI, calculated as weight in kilograms divided by height in meters squared]), preinjury functional status (living situation, ability to perform activities of daily living and instrumental activities of daily living, and use of assistive devices), admission data (length of stay [LOS], number of admissions, intensive care unit days, and ventilator days), injury severity score (ISS) data, and mechanisms of injury. The ISS, an anatomical scoring system (score range, 0-75, with higher scores indicating greater severity of injury) that provides an overall score for patients with multiple injuries, was calculated based on injuries sustained within each body system. For example, an isolated hip fracture received an ISS of 9. Data on readmission for falls after index admission and the number of falls were collected by reviewing the clinical notes from all patient encounters in the year after index admission discharge, with careful attention given to self-reported and/or guardian-reported falls and fall incidents resulting in hospitalization. Data collected were used to assign CSHA CFS scores to patients based on their preadmission health status and functional ability.

### Frailty Scoring

Frailty scores before injury were assessed by 2 of us (V.H.H. and K.S.R.) using the CSHA CFS.^8^ The CSHA CFS is a clinical opinion scale in which the scorer uses the information available to determine the patient’s frailty score. Admission history, physical examinations, consultations, initial social work notes, initial discharge planning notes (if the previous level of support was gathered), and initial physical therapy notes were evaluated by the one of us (V.H.H.) until sufficient data on medical comorbidities and the ability to perform activities of daily living were collected to determine preinjury frailty.^3^ The CSHA CFS scores ranged from 1 to 7, with a score of 1 indicating the patient was extremely active and presented with no known comorbidities and a score of 7 indicating the patient presented with a known terminal illness and needed outside help to perform daily activities (eTable in the Supplement). Frailty was defined as a score of 5 or more.

### Primary and Secondary End Points

Our primary end point was to determine whether frailty assessed using the CSHA CFS was associated with readmission for falls and the number of falls within 1 year after index admission for trauma-related injury. A fall was defined as any unintended, uncontrolled drop to the ground from a standing, sitting, or elevated position. Our secondary end points were to identify other variables associated with readmission for falls within 1 year after index admission and to assess whether frailty scores were associated with the discharge disposition after index admission.

### Statistical Analysis

Descriptive statistics were obtained, and *t* tests, Mann-Whitney tests, and χ^2^ tests were used to assess differences between the frail and nonfrail groups. All tests were 2-sided with a significance threshold of *P* < .05. Negative binomial, linear, and multivariate logistic regression analyses were performed to examine whether frailty scores were associated with LOS, discharge disposition, readmission for falls, or number of falls within 1 year after index admission, while controlling for age, sex, and BMI. Statistical analysis was performed using IBM SPSS Statistics, version 25 (IBM Corp).

## Results

A total of 827 patients aged 50 years and older who were admitted for trauma-related injury between July 1, 2010, and June 30, 2015, were identified in the University of Iowa Hospitals and Clinics trauma registry. Of those, 23 patients were excluded because they were admitted for burn injuries. The medical records of the remaining 804 patients were reviewed, and their frailty scores were calculated. The mean (SD) age of participants was 70 (13.4) years; 744 patients (93.4%) were white, and 380 patients (47.3%) were men. Among the total patients, the median number of falls was 1 (range, 0-6 falls) and the mortality rate was 3.7%, with 255 patients (31.7%) classified as frail and 549 patients (68.3%) as nonfrail. The mean (SD) ISS was 9.8 (7.9) and did not differ between frail and nonfrail patients (mean [SD], 9.5 [5.9] and 10.0 [8.6], respectively; *P* = .36) (Table 1). The population was well distributed across the frailty scale, with 0 patients presenting a score of 1, 87 patients (10.8%) a score of 2, 185 patients (23.0%) a score of 3, 277 patients (34.5%) a score of 4, 175 patients (21.8%) a score of 5, 75 patients (9.3%) a score of 6, and 5 patients (0.6%) a score of 7.

**Table 1.** Characteristics of Patients With Trauma-Related Injury

Characteristic	Nonfrail, No. (%) (n = 549)	Frail, No. (%) (n = 255)	*P* Value^a^
Age, mean (SD), y	66.2 (11.9)	79.2 (12.1)	<.001
Female	245 (44.6)	179 (70.2)	<.001
White	540 (99.4)	240 (94.5)	.89
BMI, mean (range)	27.5 (14.4-73.0)	26.3 (14.1-78.6)	.03
ISS, mean (SD)^b^	10.0 (8.6)	9.5 (5.9)	.91
Anticoagulants on admission	74 (13.5)	51 (20.1)	.01
Aspirin on admission	153 (27.8)	104 (40.6)	<.001
Hospital LOS, mean (SD), d	4.7 (6.2)	5.2 (4.1)	.06
ICU LOS, mean (SD), d	4.5 (7.1)	3.3 (2.6)	.28
History of fall	53 (9.6)	63 (24.8)	<.001
Readmission for fall	58 (10.6)	55 (21.6)	<.001
Mortality rate, %	3.6	3.9	.85

Abbreviations: BMI, body mass index (calculated as weight in kilograms divided by height in meters squared); ICU, intensive care unit; ISS, injury severity score; LOS, length of stay.

^a^ All tests were 2-sided with a significance threshold of *P* < .05.

^b^ The ISS was calculated based on injuries sustained within each body system.

Of the 255 frail patients, 179 were women (70.2%). Frail patients were significantly older (mean [SD], 79.2 [12.1] years vs 66.2 [11.9] years, respectively; *P* < .001) and had a lower BMI (mean [range], 26.3 [14.1-78.6] vs 27.5 [14.4-73.0], respectively; *P* = .03; Table 1) than nonfrail patients (n = 549). A greater proportion of frail patients were taking anticoagulants and aspirin compared with nonfrail patients (51 [20.1%] vs 74 [13.5%]; *P* = .01 and 104 [40.6%] vs 153 [27.8%], respectively; *P* < .001). In addition, the percentages of frail patients presenting to the hospital with a history of falls and readmitted for falls after index admission were higher than those of nonfrail patients (63 [24.8%] vs 53 [9.6%] and 55 [21.6%] vs 58 [10.6%], respectively; *P* < .001).

**Figure 1.**
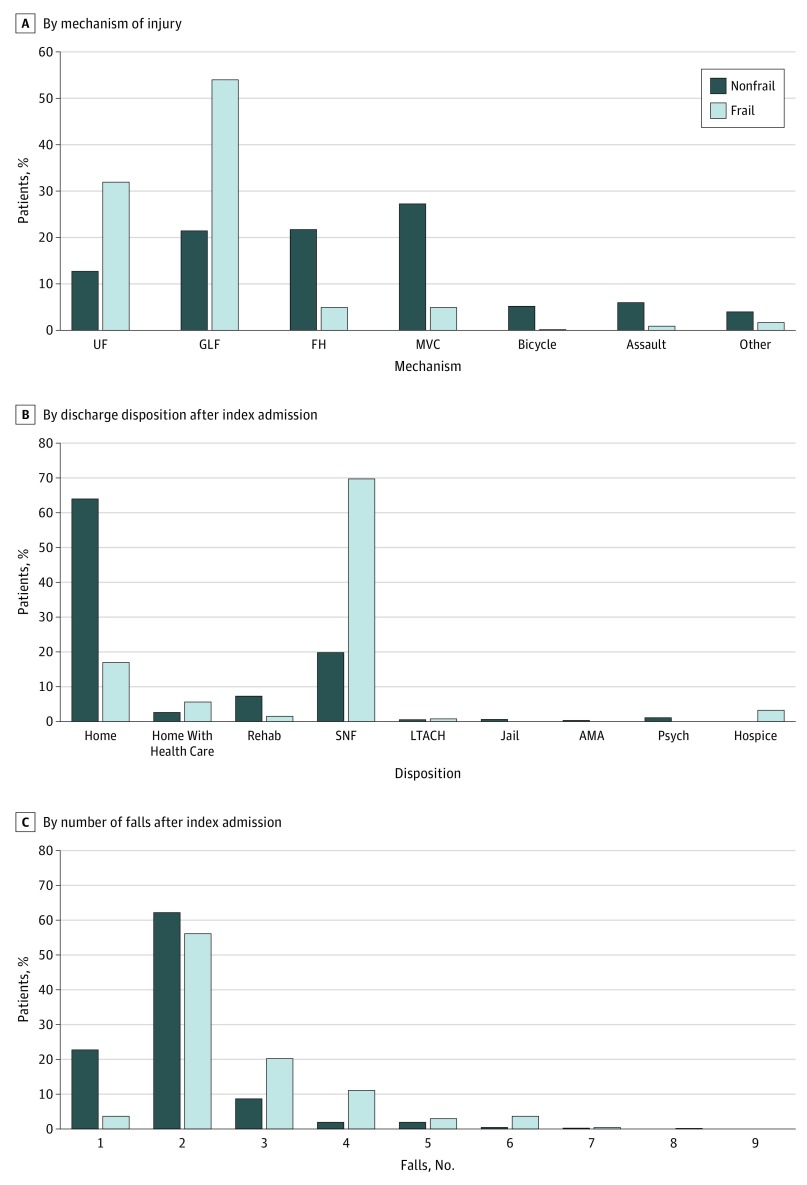
Distribution of Frail and Nonfrail Patients With Trauma-Related Injury UF indicates unspecified fall; GLF, ground-level fall; FH, fall from height; MVC, motor vehicle collision; rehab, rehabilitation facility; SNF, skilled nursing facility; LTACH, long-term acute care hospital; AMA, left against medical advice; and psych, psychiatric care facility.

Frail patients were more likely to present to the hospital with either an unspecified fall (82 [32.2%]) or a ground-level fall (GLF; 138 [54.1%]) as the initial mechanism of injury (Figure 1A). In comparison, the nonfrail cohort was more evenly distributed, with GLFs (119 [21.7%]), falls from heights (121 [22.0%]), and motor vehicle collisions (151 [27.5%]) composing most of the mechanisms of injury.

A larger proportion of frail patients (179 [69.8%]) was discharged to a skilled nursing facility compared with nonfrail patients (111 [20.2%]), and only 42 frail patients (17.1%) were discharged to the home compared with 339 nonfrail patients (64.1%) (Figure 1B). In addition, a larger proportion of frail patients fell 2 or more times after index admission compared with nonfrail patients (56 [22.0%] vs 51 [9.3%], respectively) (Figure 1C).

**Figure 2.**
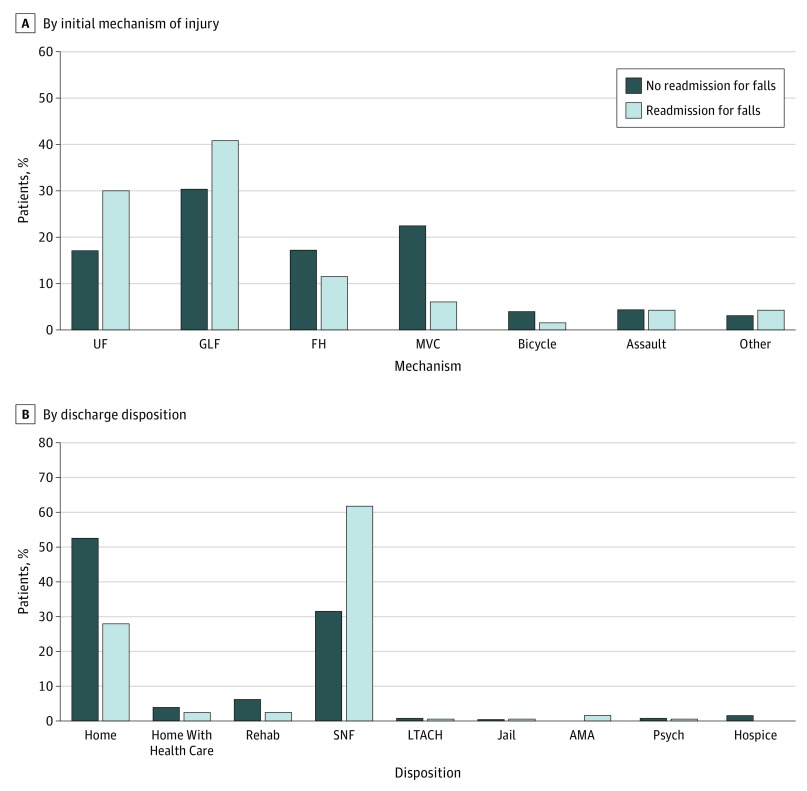
Distribution of Patients With Trauma-Related Injury Who Were Readmitted for Falls UF indicates unspecified fall; GLF, ground-level fall; FH, fall from height; MVC, motor vehicle collision; rehab, rehabilitation facility; SNF, skilled nursing facility; LTACH, long-term acute care hospital; AMA, left against medical advice; and psych, psychiatric care facility.

Most patients readmitted for falls originally presented to the hospital with injuries from unspecified falls or GLFs (33 [30.0%] and 45 [40.9%], respectively) (Figure 2A). In addition, a greater proportion of patients who were readmitted for falls were more likely to be discharged to an SNF (68 [61.2%]) compared with patients who were not readmitted for falls, who were largely discharged to the home with no assistance (350 [52.7%]) (Figure 2B).

A binary logistic regression analysis, controlled for age, BMI, sex, anticoagulant or aspirin use, and GLF injury at index admission, indicated that frailty was associated with readmission for falls within 1 year after index admission (odds ratio [OR], 2.26; 95% CI, 1.39-3.66; *P* = .001) compared with nonfrailty. Age, BMI, sex, anticoagulant or aspirin use, and GLF injury at index admission were not associated with readmission for falls. Additional analyses were performed for injury patterns that could be potential confounders, which included traumatic brain injuries, femur fractures, hip/pelvis fractures, and lower extremity fractures. The results suggested that frailty remained associated with readmission for falls within 1 year after index admission (OR, 1.49; 95% CI, 1.19-1.86; *P* = .001). None of these injuries was significantly associated with readmission for falls within 1 year after index admission.

Frailty was not associated with the mortality rate (3.7% overall) in this population. A multinomial logistic regression analysis, controlled for age, sex, BMI, and GLF injury at index admission, indicated that frailty was associated with discharge disposition to the home with health care (OR, 4.82; 95% CI, 2.10-11.01; *P* < .001), to an SNF (OR, 5.47; 95% CI, 3.40-8.80; *P* < .001), and to a hospice care facility (OR, 8.47; 95% CI, 2.09-34.42; *P* = .003) compared with discharge to the home with self-care. In this model, female patients with trauma-related injury were more likely to be discharged to the home with health care (OR, 2.31; 95% CI, 1.03-5.17; *P* = .04) and to an SNF (OR, 2.54; 95% CI, 1.64-3.91; *P* < .001) compared with discharge to the home. In addition, index admission for a GLF injury was associated with discharge disposition to an SNF (OR, 2.44; 95% CI, 1.55-3.84; *P* < .001) and to a hospice care facility (OR, 3.64; 95% CI, 1.01-13.11; *P* = .048) compared with discharge to the home with self-care.

A negative binomial regression analysis, controlled for age, BMI, sex, and GLF injury at index admission, indicated that frailty was associated with a greater number of falls within 1 year after index admission (OR, 1.32; 95% CI, 1.04-1.67; *P* = .02) compared with nonfrailty. Additional analyses were performed for injury patterns that could be potential confounders, which included traumatic brain injuries, femur fractures, hip/pelvis fractures, and lower extremity fractures. The results suggested that frailty remained associated with the number of falls within 1 year after index admission (OR, 1.49; 95% CI, 1.19-1.86; *P* = .001) compared with nonfrailty. None of these injuries were associated with the number of falls within 1 year after index admission.

Independent assessment of each frailty score indicated that frailty scores of 5 and 6 were associated with a greater number of falls (OR, 1.84; 95% CI, 1.19-2.86; *P* = .006 and OR, 1.83; 95% CI, 1.10-3.02; *P* = .02, respectively) compared with all other frailty scores. Among the total population of patients with trauma-related injury, frailty was not associated with hospital LOS compared with nonfrailty (mean [SD], 5.2 [4.1] days vs 4.7 [6.2] days, respectively; *P* = .06).

Ground-level falls were the main mechanism of injury among the total population, with 257 patients (32.0%) presenting with a GLF injury at index admission. In addition, 138 frail patients (54.0%) presented with a GLF injury at index admission, and only 119 nonfrail patients (21.7%) were admitted for a GLF injury (Figure 1A). Frail patients with a GLF injury at index admission were significantly older (mean [SD], 79.9 [11.9] years vs 74.1 [12.1] years, respectively; *P* < .001) and stayed in the hospital longer (mean [SD], 5.2 [3.9] days vs 4.3 [3.1] days, respectively; *P* = .04) than nonfrail patients with a GLF injury at index admission (Table 2), and the rate of frail patients presenting to the hospital with a history of falls was higher than that of nonfrail patients (37 [26.8%] vs 19 [16.0%], respectively; *P* = .04). However, the rate of readmission for falls after index admission was not significantly different between the frail and nonfrail groups.

**Table 2.** Characteristics of Patients Admitted for a Ground-Level Fall at Index Admission

Characteristic	Nonfrail, No. (%) (n = 119)	Frail, No. (%) (n = 138)	*P* Value^a^
Age, mean (SD), y	74.1 (12.1)	79.9 (11.9)	<.001
Female	72 (60.5)	99 (71.7)	.06
White	110 (92.4)	131 (94.9)	.28
BMI, mean (range)	26.5 (15.5-73.0)	25.8 (14.3-59.0)	.63
ISS, mean (SD)	9.9 (4.4)	9.3 (4.7)	.81
Hospital LOS, mean (SD), d	4.3 (3.1)	5.2 (3.9)	.04
ICU LOS, mean (SD), d	3.1 (4.2)	3.3 (2.8)	.89
History of fall	19 (16.0)	37 (26.8)	.04
Readmission for fall	20 (16.8)	25 (18.1)	.78
Mortality rate, %	4.2	2.9	.57

Abbreviations: BMI, body mass index (calculated as weight in kilograms divided by height in meters squared); ICU, intensive care unit; ISS, injury severity score; LOS, length of stay.

^a^ All tests were 2-sided with a significance threshold of *P* < .05.

Frailty was not associated with readmission for falls (among patients with a GLF injury only; OR, 0.90; 95% CI, 0.46-1.76; *P* = .76) or mortality (OR, 1.98; 95% CI, 0.49-7.95; *P* = .33) in a multivariate logistic regression analysis controlled for age and sex. Frailty was also not associated with the number of falls within 1 year after index admission (OR, 0.89; 95% CI, 0.63-1.24; *P* = .48). However, frailty was associated with discharge to the home with health care (OR, 12.34; 95% CI, 2.74-55.59; *P* = .001), to an SNF (OR, 9.66; 95% CI, 4.30-21.72; *P* < .001), and to a hospice care facility (OR, 31.87; 95% CI, 3.40-298.32; *P* = .002) compared with discharge to the home with self-care. A linear regression analysis controlled for age and sex indicated that frail patients stayed in the hospital an average of 1.2 days longer than nonfrail patients (95% CI, 0.32-2.10 days; *P* = .008).

## Discussion

Although aging has been associated with risk factors for falls, such as changes in gait and balance, increased inactivity, severe chronic conditions, and increased prescription medication use,^13^ age alone has not been associated with physiological fitness in the elderly population. Thus, many scales have been developed to measure patient frailty. In our study, use of the CSHA CFS indicated that frail patients with trauma-related injury were more likely to be readmitted for falls after index admission and to be discharged to an SNF compared with nonfrail patients. In addition, frail patients who were admitted for a GLF injury at index admission were more likely to have a history of falls, an increased hospital LOS, and discharge disposition to an SNF.

In our study, frailty was associated with discharge disposition to an SNF among patients with trauma-related injury, which is consistent with previous studies that used the CSHA CFS among patients with burn injuries^11^ and trauma-related injuries who were older than 50 years.^3^ Previous studies examining discharge disposition and mortality suggested a 66% increased risk of postdischarge mortality in patients who were not discharged to the home^14^ and up to a 3-fold increase in the risk of death in the year after trauma-related injury among patients discharged to an SNF compared with those discharged to the home without assistance.^15^

Our study results also indicated that frailty was associated with readmission for falls after index admission. In contrast, a study by Chua et al^16^ indicated that frailty status determined using the CHSA CFS was not associated with a higher rate of rehospitalization within 6 months after hospital discharge. However, this discrepancy might be explained by the differences in inclusion and exclusion criteria between the studies. Chua et al^16^ excluded patients younger than 70 years, patients with acute functional decline whose frailty status had acutely changed, and patients who were transferred to their facilities from outside institutions. Our study included all patients aged 50 years and older. Furthermore, the discrepancy may be owing to a difference in the time frame of readmissions examined in our study (6 months) compared with the study by Chua et al^16^ (12 months).

Previous studies of falls indicated that community-dwelling older adults experienced 1 or more falls per year.^17,18,19,20^ Among the patients with trauma-related injury in our study, frailty scores were associated with a higher number of falls in the year after index admission, with CSHA CFS scores of 5 and 6 associated with the highest number of falls within that year. When we narrowed our analysis to patients who presented with a GLF injury at index admission, we noticed that frail patients admitted for a GLF injury were older and had a longer hospital LOS compared with nonfrail patients. In an analysis controlled for age and sex, we found that frail patients stayed in the hospital a mean of 1.2 days longer for their initial fall compared with nonfrail patients. These results are in line with those by Juma et al,^21^ who reported that frailty scores were associated with LOS among elderly patients in an acute care unit. Taken together, our data suggest that frailty scores determined using the CSHA CFS may be used to assess fall risk in patients with trauma-related injury and to ascertain the hospital LOS of patients admitted for a GLF injury.

The proportion of frail patients who were admitted for a GLF injury and had a history of falls was larger than that of nonfrail patients; however, frailty was not associated with readmission for falls when controlling for age and sex. This finding is different than our analysis of all patients with trauma-related injury, which suggested that frailty is associated with readmission for falls in the year after index admission. This discrepancy is likely multifactorial because an initial SNF discharge disposition may have been associated with a decrease in the fall risk among frail patients admitted for a GLF injury, bringing it near the threshold for nonfrail patients admitted for a GLF injury. Furthermore, this discrepancy could be associated with variations in the severity of trauma-related injury between those in the all-trauma analysis and those admitted for a GLF injury at index admission. Finally, using the Healthcare Cost and Utilization Project’s Nationwide Readmissions Database, Hoffman et al^22^ reported that fall-related injuries were the second leading diagnosis for readmission among patients admitted for fall-related injuries at index admission, suggesting that falls at index admission are associated with a greater likelihood of a fall requiring readmission. Based on these results and the observation that, in our GLF cohort, frail patients who experienced a GLF injury were more likely to have a history of falls, one could consider that frailty and falls are associated. Thus, we may have controlled for frailty by narrowing our analysis to only GLF injuries, which may explain why no association between frailty and readmission for falls was found.

### Limitations

Our study had several limitations. The data were acquired through retrospective medical record reviews, which introduced a risk of bias owing to poor documentation or missing data. In addition, there was a risk of bias in our calculation of frailty scores and, therefore, in their ability to establish associations with outcomes, as we used documentation from a variety of sources to calculate frailty scores and may have been aware of the outcomes. We attempted to account for this potential bias by determining our scores using only admission documentation and by blinding ourselves to the outcomes while scoring occurred. The possibility of an association between higher frailty scores and the need for discharge to an SNF after hospitalization also inherently existed. Most patients, even those with high frailty scores, were not residing in an institution before their hospitalization, and discharge to an SNF has been reported to be associated with an increased risk of death,^23^ which makes the finding that patients who are more frail at admission are more likely to be discharged to an SNF compelling, although somewhat expected.

Data were collected from the electronic medical records of patients who originally presented to the University of Iowa Hospitals and Clinics with trauma-related injury. The number of true falls may have been underestimated in our study for several reasons. First, patients may have presented to an outside hospital or health care institution for subsequent falls in the year after their discharge, and those falls would not have been included in our patient analysis. Second, falls may have occurred and been managed in institutions, such as long-term acute care hospitals, SNFs, or nursing homes, and those patients may not have presented back to our institution. Third, our study used patient and guardian self-reporting of subsequent falls, which introduced reliability concerns. Another limitation was the fact that our study did not have sufficient power to assess whether frailty was associated with the likelihood of mortality within our patient population, as this association was not a primary end point. In addition, we only captured in-hospital mortality because the hospital was not always notified of a patient’s death after discharge.

Despite the limitations, our data suggest that use of the CSHA CFS to assess frailty may also be helpful in evaluating the likelihood of readmission for falls and number of falls as well as the likelihood of discharge disposition to an SNF in patients with trauma-related injury. Such information may also be useful in referring patients to fall prevention programs. Among patients with a GLF injury, frailty assessment was associated with hospital LOS and discharge disposition to an SNF. Such information could be used to better inform patients and their families about their possible hospital course and costs and to make them aware that the patients may not be able to return to independent living.

## Conclusions

Fall prevention is essential, as the health care costs associated with falls and readmissions from fall-related injury are high. As the US population ages, the number of falls and the associated costs are likely to increase. Evidence-based fall prevention and physical activity programs designed specifically for older adults have been developed. However, most of these programs have not been as successful as they could have been owing to a lack of screening for fall risk in primary care settings. Our study suggests that frailty assessment at hospital admission may provide an effective tool to evaluate fall risk in patients with trauma-related injury aged 50 years and older. In addition, frailty was associated with discharge disposition to an SNF and hospital LOS, and frailty status may provide useful information for assessing potential health care costs. Future studies are needed to examine the utility of frailty assessment in patients with trauma-related injury.

## Notes

Supplement. **eTable.** Canadian Study of Health and Aging Clinical Frailty Scale
