# Supplementary material for: Association of Clinical Frailty Scores With Hospital Readmission for Falls After Index Admission for Trauma-Related Injury
Source: JAMA Netw Open. 2019 Oct 2;2(10):e1912409. doi: 10.1001/jamanetworkopen.2019.12409 (PMC6777261; doi:10.1001/jamanetworkopen.2019.12409)
Supplement: Supplement. — eTable. Canadian Study of Health and Aging Clinical Frailty Scale [file jamanetwopen-2-e1912409-s001.pdf]

Supplementary Online Content

Hatcher VH, Galet C, Lilienthal M, Skeete DA, Romanowski KS. Association of clinical frailty scores with hospital readmission for falls after index admission for trauma-related injury. *JAMA Netw Open*. 2019;2(10):e1912409. doi:10.1001/jamanetworkopen.2019.12409

eTable. Canadian Study of Health and Aging Clinical Frailty Scale

This supplementary material has been provided by the authors to give readers additional information about their work.

© 2019 Hatcher VH et al. *JAMA Network Open*.

eTable. Canadian Study of Health and Aging Clinical Frailty Scale

**Frailty definition**	**CSHA CFS score**	**Clinical definition**
**Non Frail**	**1**	The patient is extremely active (and this is noted in the history) and presented with no known comorbidity.
	**2**	The patient presented with no known comorbidity
	**3**	The patient presented either with one or more known comorbidity, all controlled by medication
	**4**	The patient presented with at least one known comorbidity controlled by multiple medications, two to four comorbidities not controlled by medication, or five or more comorbidities.
**Frail**	**5**	The patient required assistive devices to ambulate or required oxygen supplementation.
	**6**	The patient required outside help to perform daily activities.
	**7**	The patient presented with a known terminal illness and outside help to perform daily activities.

© 2019 Hatcher VH et al. *JAMA Network Open*.
